# Response to Leopoldo Palma. Comments on Ekino *et al.* Cloning and Characterization of a Unique Cytotoxic Protein Parasporin-5 Produced by *Bacillus thuringiensis* A1100 Strain. *Toxins* 2014, *6*, 1882–1895

**DOI:** 10.3390/toxins7124866

**Published:** 2015-11-27

**Authors:** Keisuke Ekino, Shiro Okumura, Tomoyuki Ishikawa, Sakae Kitada, Hiroyuki Saitoh, Tetsuyuki Akao, Takuji Oka, Yoshiyuki Nomura, Michio Ohba, Takashi Shin, Eiichi Mizuki

**Affiliations:** 1 Department of Applied Microbial Technology, Faculty of Biotechnology and Life Science, Sojo University, 4-22-1 Ikeda, Kumamoto 860-0082, Japan; oka@bio.sojo-u.ac.jp (T.O.); nomura@bio.sojo-u.ac.jp (Y.N.); shin@bio.sojo-u.ac.jp (T.S.); 2 Biotechnology and Food Research Institute, Fukuoka Industrial Technology Center, 1465-5 Aikawa-machi, Kurume, Fukuoka 839-0861, Japan; sokumura@fitc.pref.fukuoka.jp (S.O.); tishikawa@fitc.pref.fukuoka.jp (T.I.); saitou@fitc.pref.fukuoka.jp (H.S.); akao@fitc.pref.fukuoka.jp (T.A.); emizuki@fitc.pref.fukuoka.jp (E.M.); 3 Department of Bioscience and Bioinfomatics, Kyushu Institute of Technology, Iizuka, Fukuoka 820-8502, Japan; kitada@bio.kyutech.ac.jp; 4 Graduate School of Agriculture, Kyushu University, Fukuoka 812-8581, Japan; gunka@yk2.so-net.ne.jp

I appreciate the thoughtful comments from Dr. Leopoldo Palma [1] on our research about cytotoxic protein parasporin-5 produced by *Bacillus thuringiensis* (BT) A1100 strain [2]. First of all, the term parasporin is defined as “*Bacillus thuringiensis* and related bacterial parasporal proteins that are non-hemolytic but capable of preferentially killing cancer cells” [3,4]. The definition does not refer to insecticidal activity. Mizuki *et al.* carried out a large-scale screening of BT strains for the new biological activity, that is, cytocidal activity to human cancer cells with non-hemolytic activities [5]. In this study, 1744 BT strains were investigated not only for cytocidal activity but also for insecticidal activities against 11 species of five orders (Lepidoptera, Diptera, Orthoptera, Dictyoptera, and Isoptera). As a result, 42 cytocidal BT strains, parasporin producers, have been discovered, all of which have non-insecticidal activities as far as tested. Further, the A1100 strain in our study was not toxic to the above 11 species. However, the insecticidal activity that we have studied was limited. Based on the above definition of parasporin, we do not deny the possibility that parasporins have insecticidal activity. Known parasporins might have insecticidal activity and novel insecticidal parasporins may be discovered. Further interesting research will clarify this unknown parasporin world.
